# Measuring health inequities in low and middle income countries for the development of observatories on inequities and social determinants of health

**DOI:** 10.1186/s12939-016-0297-9

**Published:** 2016-01-19

**Authors:** German Guerra, Elis Borde, V. Nelly Salgado de Snyder

**Affiliations:** National Institute of Public Health, Mexico (INSP), Av. Universidad No. 655 Colonia Santa Maria Ahuacatitlán, C.P. 62100 Cuernavaca, Morelos Mexico; Centro de Estudos, Políticas e Informações sobre Determinantes Sociais da Saúde, Escola Nacional de Saúde Pública Sergio Arouca, Fundação Oswaldo Cruz, Rua Leopaldo Bulhões 1480, Rio de Janeiro, RJ 21041-210 Brazil; National Institute of Public Health, Mexico (INSP), Av. Universidad No. 655 Colonia Santa Maria Ahuacatitlán, Cuernavaca, Morelos Mexico

**Keywords:** Social determinants of health, Health status disparities, Methods, Data collection

## Abstract

**Background:**

Almost seven years after the publication of the final report of the World Health Organization’s Commission on Social Determinants of Health (CSDH), its third recommendation has not been attended to properly. Measuring health inequities (HI) within countries and globally, in order to develop and evaluate evidence-based policies and actions aimed at the social determinants of health (SDH), is still a pending task in most low and middle income countries (LMIC) in the Latin American region. In this paper we discuss methodological and conceptual issues to measure HI in LMIC and suggest a three-stage methodology for the creation of observatories on health inequities (OHI) and social determinants of health, based on the experience of the Brazilian Observatory on Health Inequities (BOHI) that has been successfully operating since 2010 at the Fundação Oswaldo Cruz (FIOCRUZ).

**Methods:**

A three-stage methodology for the creation of an OHI was developed based on a literature review on the following topics: SDH, HI measurement, and the process of setting-up of health observatories; followed by semi-structured interviews with key informants from the BOHI. We describe the three stages and discuss the replicability of this methodology in other Latin American countries. We also carried out a search of suitable national information systems to feed an OHI in Mexico, along with an outline of the institutional infrastructure to sustain it.

**Results:**

When implementing the methodology for an OHI in LMIC such as Mexico, we found that having strong infrastructure of information systems for measuring HI is required, but not sufficient to build an OHI. Adequate funding and intersectoral network collaborations lead by a group of experts is a requirement for the consolidation and sustainability of an OHI in LMIC.

**Conclusion:**

According to the described methodology, and the available information systems on health, the creation of an OHI in LMIC, particularly in Mexico, is plausible in the near future. However, institutional support (in academic, financial, and policymaking terms) is essential to materialize such needed instance, thus locally contributing to attain health equity.

## Background

Almost seven years after the publication of the final report of the World Health Organization’s Commission on Social Determinants of Health (CSDH), its third recommendation has not been attended to properly. Measuring health inequities (HI) within countries and globally, in order to develop and evaluate evidence-based policies and actions aimed at the social determinants of health (SDH) [1], is still a pending task in most low and middle income countries (LMIC) in the Latin American region. In this paper we discuss methodological and conceptual issues to measure HI in LMIC and suggest a three-stage methodology for the creation of observatories on health inequities (OHI) and social determinants of health, based on the experience of the Brazilian Observatory on Health Inequities (BOHI) that has been successfully operating since 2010 at the Center for Studies, Policies and Information on Social Determinants of Health (CEPI-DSS) in Fundação Oswaldo Cruz (FIOCRUZ).

### Associations between inequality, inequity, health and measurement

The recent awareness for measuring HI at the global level owes its relevance to the publication of the report of the World Health Organization’s Commission on Social Determinants of Health (CSDH) in 2008. Nonetheless, the scientific discussion of how to comprehensively and adequately measure HI has aged in its theoretical, empirical, philosophical and technical realms, and it can be traced back to a central question: Why are health disparities or inequalities persistent between population groups?

Given the fact that health disparities^1^ manifest in worse health status for disadvantaged population groups, for instance in those with low income or education levels [2], for measuring purposes it is generally accepted that health inequality indicators are considered an empirical evidence of health inequities [3]. This assumption, however, lacks the ethical arguments inherently contained in the concept of equity as a principle of social justice [4, 5].

When assessing health inequalities there is a need to question whether they derive from social inequities –understood as the imbalanced distribution of power, prestige and resources that result in disadvantages among population groups– that have a direct or indirect impact on health status. In this concern, the SDH or the “the conditions in which people are born, grow, live, work and age [due to the] circumstances [that] are shaped by the distribution of money, power and resources at global, national and local levels” [6], including the health system; provide a powerful framework for the assessment of health inequalities, which are unfair and avoidable [7].

According to the conceptual framework of SDH by the CSDH, the differences in health status among population groups derive from the interplay of two sets of social determinants: the ‘structural social determinants of health’, which include the socioeconomic and political context and the socioeconomic position (education, occupation and income); and the ‘intermediary social determinants of health’, namely the material circumstances (living and work conditions) and behavioral and psychosocial factors [7].

From a traditional public health or classical epidemiology perspective, health inequalities are expressed as stratified indicators (i.e. mortality rates by education or income levels) that work as proxies of the intermediary SDH. Although these types of measures are crucial for describing health status differences in population groups, they are not frequently linked to the structural SDH that explain the persistence of such health disparities. Some exceptional examples that try to link structural and intermediary SDH exist in LMIC [8, 9] yet, in these studies, the discussion of health inequalities as a result of the unfair distribution of social justice remains limited.

Technically, the linkage between the ethical-philosophical and the empirical dimensions of equality into coherent and integrative indicators is one of the biggest challenges in measuring HI because it implies the operationalization of a concept as complex as equity^2^. A definition that aids this linkage refers to health equity as “the absence of systematic disparities in health (or in the major social determinants of health) between social groups who have different levels of underlying social advantage/disadvantage—that is, different positions in a social hierarchy” [4]. This definition considers the measurement of health disparities as indispensable for the assessment of HI. Therefore, a previous step for measuring and monitoring HI as recommended by the CSDH, is to create and strengthen health information systems at the national level [10] that would serve as the empirical basis for linking health equality to equity as a social justice principle.

### National Health Observatories

The challenges of monitoring HI are not only centered on the issue of measurement. Also, there is a need for an infrastructure embedded at the institutional level that is assigned to the tasks of: a) gathering health and healthcare-related data; b) processing data as indicators; c) interpreting results in the local context and; d) disseminating results for diverse audiences, including general public, civil society organizations, non-government organizations (NGO), decision-makers at the policy level, and researchers and academia, among others. Such infrastructure consolidates in the health observatories, which in a broader sense, are institutional platforms where health data is processed and transformed into value-added goods for public use, such as open access datasets, printed and digital media (infographics, bulletins), and informative documents on nationally relevant health topics.

An operational definition of a health observatory is provided by Gattini as “a policy-oriented virtual based national center aimed at performing systematic and ongoing observation on relevant issues about population health and health systems, in support of effective and evidence-based health policy, planning, decision-making and action in public health and health systems. The ultimate goal is to contribute to the preservation and improvement of health of the population, including the reduction of inequalities” [11]^(p.11)^. In this definition, there is an emphasis on *observation* as the act of focusing the attention on ongoing events with the purpose of registering, analyzing or predicting their outcomes in a contextual manner. Under this perspective, observing the health status of a population during a specific period of time implies not only registering epidemiological or health system related events, but it also involves the capacity of analyzing and relating them to their social determinants, and suggesting entry points for health policy formulation. For these reasons, a health observatory –and specifically an observatory for HI– constitutes a complex institutional platform that is built upon the interaction of diverse social actors and sectors that have the common goal of observing trends in HI, identifying strategies to modify SDH, and recommending specific actions that respond to the social challenges that are associated with health disparities.

### The Brazilian Observatory on Health Inequities (BOHI)

The creation of the WHO Commission on SDH (CSDH) in 2005 and particularly the creation of the Brazilian National Commission on Social Determinants of Health (BCSDH) in March 2006 gave new impetus to a long tradition of research and action to fight disparities in Brazil, forging concrete initiatives directed at monitoring, measuring and assessing HI [12]. One of such initiatives in 2011 was the creation of the *Observatório sobre Iniquidades em Saúde*/the Brazilian Observatory on Health Inequities (BOHI) at the CEPI-DSS. The observatory efforts concentrated on implementing the recommendations of the BCSDH and promoting research, policies and information on SDH, as recommended by the 2011 Rio Political Declaration.

The BOHI was foreseen to serve as an “open space for information, reference, dialog and communication for several key actors from civil society and government organizations whose role is the definition and implementation of social policies aimed at tackling health inequities by addressing and acting on the social determinants of health” [13]. The BOHI was built upon the *Rede Interagencial de Informações Para a Saúde* or the Interagency Network for Health Information (RIPSA), a strategy launched in 1995 by the Pan American Health Organization and the Brazilian Ministry of Health with the purpose of bringing together the scientific community to discuss, analyze and disseminate information for understanding the health of Brazilians, and reach consensus on concepts, methodologies and use of information systems for the creation of health disparities indicators [14].

The design of the BOHI involved first an analysis of 51 health observatories from different countries, with various levels of data aggregation (national, regional, subregional and county levels) which helped to set the scope and characteristics of the BOHI [15]. Currently, the BOHI is hosted by CEPI-DSS and operates together with an online portal on SDH which pools research results, interviews, expert opinions and experiences and provides the basis for periodic reports on HI, supporting the design of health policies at the national, regional and sub-regional levels in Brazil (http://dssbr.org/site/). BOHI’s main objective is “to monitor the trends of HI and their determinants in Brazil in order to support policies and programs developed by government and non-government organizations that aim to tackle HI" [15, 16].

The BOHI is a project of great relevance in Brazil, and like any complex endeavor, it faces some important challenges, one of them being sufficient funding, as mentioned by its founders:

*“The is a need of allocation of sufficient funds to retain an adequate number of professionals to sustain the observatory, which requires stronger commitment from decision makers and institutional guarantees”*

Former General Coordinator of CEPI-DSS

Furthermore, there has been some concern that the BOHI is functioning as a database rather than an observatory producer of critical evidence for policy making, assessment, accountability and the dissemination of periodic publications using the online portal infrastructure and other communication channels (personal communication with current CEPI-DSS General Coordinator. November 2015).

The BOHI experiences described above correspond to what Gattini called the "empirical basis" for the development of health observatories, and it is a prerequisite for creating health observatories in LMIC, including Mexico [11].

## Methods

In this section we provide the reader with an overview of the procedure used to develop a three-stage methodology for the creation of an OHI, firstly addressing the empirical basis and requirements, followed by a discussion of the replicability of this methodology in LMIC (particularly in Mexico) where health data availability tends to be sparse, scarce or inconsistent [17].

The following overview is based mainly on two activities. First, a scientific literature review using the following search terms: *health inequalities*, *measurement, methods and theories*, *social determinants of health* and *Latin America.* The search was carried out in two search engines: PubMed (United States National Library of Medicine) and BIREME (Pan American Health Organization’s Virtual Health Library).

The search included also grey/unpublished literature on the following two topics: *health observatories* and *health inequities*, which have not yet being indexed as search terms at either BIREME or PubMed. Therefore this search was conducted in Google Scholar, the CEPI-DSS online portal on SDH, and the SDH-Net online repository (http://tie.inspvirtual.mx/portales/sdhnet/sdh-net/). The searches were made during March-April 2014.

Based on the literature review two topic guides were designed and used to conduct three semi-structured interviews with key informants involved in the development of the BOHI: a) two technical consultants to CEPI-DSS, and; b) a former General Coordinator of the CEPI-DSS. The interviews were carried out in April 2014. Both activities (literature review and interviews) lead to the systematization of a three-stage methodology for the development of OHI in LMIC using the empirical basis guidelines suggested by Gattini [11].

### Empirical basis for the creation of an OHI

The creation of an OHI calls first for identifying and gathering some basic elements. First, an observatory is built upon (and ultimately depends on) the existing primary sources of sociodemographic information, such as population census, geographical information systems or vital statistics. It also uses more specific sources like epidemiological surveillance, nutritional status, and health system performance surveys. The degree of success for implementing an OHI depends on strong and accessible health information systems, although, robust information systems are not the only requirement for its creation. Developing an OHI also calls for procedural frameworks and policies for accessing the sources of information [11].

A second element is the installed capacity in areas of government, such as local and national level ministries of health, social development, etc., to generate periodic reports on health and health-related issues that may be relevant for decision-making. This capacity is linked to a third requirement, which is the participation of decision-makers or their advisory groups.

*“Policymakers should not only be able to design and apply evidence-based health policies, but also to communicate with other key actors, enabling social participation. For instance, during advocacy processes there are windows of opportunities for implementing initiatives to act on social determinants, such as the development of an observatory…[policy makers] must act as interlocutors with other sectors, including other government bodies, academia, civil society, etc.”*

Former General Coordinator of CEPI-DSS

Table 1 summarizes the three aforementioned components for the empirical base of a HI observatory.

**Table 1:** Empirical basis for an observatory on health

COMPONENT I	COMPONENT II	COMPONENT III
Primary and specific information systems	Installed capacity at government organizations for publishing periodic reports on health and health-related issues	Installed capacities at the decision-makers level
Census; demographic and health surveys; administrative records; vital statistics; epidemiological surveillance, etc.	Public and periodical issuing of: Executive summaries or panoramic overviews of population health status; reports on health system performance; reports of evaluations of implemented policies, etc.	Capacities on evidence-based policy and decision-making
		Intersectoral dialog capacity
CROSS-CUTTING COMPONENTS		
Institutional framework for accessing sources of information; social participation and collaborative networks		

Source: Based on information from Gattini, 2009 [11]

According to the experts, it is noteworthy that for the correct design, implementation and operation of an OHI:

*“…it is necessary that the interested sectors work under a collaborative network scheme with strong leadership and coordination from an experts’ group on health disparities and inequities”*

BOHI Former Consultant #2

Such group of experts would be ideally integrated by high-level representatives from interested sectors –preferably including (but not limited to): *producers* of health and health-related data (General Managers, Directors or Coordinators from information systems bureaus); *consumers* of health and health-related data for equity analysis (academia; NGO; government agencies); and *decision-makers* of health policies [14].

Finally, it should be mentioned that there are different types of observatories, depending on the available resources:

*“The observatory of health inequities mirrors the installed capacities and infrastructure of the institutions; but also reflect priorities, institutional frameworks and diverse types of proposals for its development”*

BOHI Former Consultant #2

We highlight three examples of this. A first type of observatory has a regional or global focus (geographically limited), an integral structure for gathering and analyzing data, and a production of periodic reports on health status accompanied by different strategies for their dissemination and knowledge translation to diverse audiences (i.e. policy-makers; civil society, etc.). An example of this is the *European Observatory on Health Systems and Policies* [18].

A second example is an observatory such as the BOHI where national health data from different sources is gathered and processed under the lens of SDH, providing an open source of information for analyses on HI that can complement the analyses and reports published elsewhere.

Finally, a third example of an observatory is the Regional Observatory of Collective Health, Environment and Society from the *Universidad Andina Simón Bolivar* in Ecuador [19] and the PAHO/WHO Portal for the Equity List and Knowledge Network [20]. These observatories primarily seek to disseminate information on HI, supporting and promoting evidence- and experience-based action on SDH by facilitating access to scientific evidence and good-practice case studies.

### Development of indicators for an OHI

A health indicator can be defined as a synthetic measure that contains relevant information on the health status of population groups and their living conditions [17]. The construction of an indicator is based on their method of calculation, from simple to complex. Instances of such indicators include among others: number of cases (counting the accumulated cases of deaths, births, etc. over a period of time); mortality or fertility rates (ratio of number of cases in relation to the population exposed to the risk of the event, multiplied by a factor); and probability of death and years of life expectancy at age “x” (actuarial methods for life table) [14, 21].

However, defining HI indicators is a more complex task because it also involves assessing the social circumstances that determine the differences between the groups. Therefore, we suggest that HI indicators should have an *associative power* for health status and social conditions. This can be done by choosing stratifying variables to health indicators (i.e. years of schooling (stratifying variable) associates with maternal mortality (health indicator); precarious work conditions (low income, unhealthy working environments, etc.) relate to worst self-perceived health conditions or higher relative risks of occupational diseases.

Additionally, HI indicators should contain an *explanatory power* on existing gaps and gradients between population groups. Once a health indicator is stratified it should allow for questioning or hypothesizing why the expressed disparity is unfair and avoidable [22]. This is done through a process of consensual decision of both, stratifying variables and health indicators:

*“The selection of indicators is preceded by extensive discussions within the experts group where an assessment of each health indicator is carried out in order to determine how adequately such indicator reflects the health status of population groups, or the social conditions that shape health inequities.”*

BOHI Former Consultant #1

The inclusion of an indicator in a core set of indicators is determined by its *validity* (capacity to measure what it intends to measure); *sensitivity* (ability to detect the analyzed phenomenon); and *specificity* (ability to only detect the analyzed phenomenon). Indicators should have these attributes: they should be obtainable with available data (*measurability*); they should answer to health priorities (*relevance*); and they should be justifiable in terms of cost (*cost-effectiveness*). Finally, “the selection of the core set of indicators –and their levels of geographical disaggregation– must be adjusted to the availability of information systems, data sources, resources, priorities and needs in each region” [17]^p.4^.

**Fig. 1:**
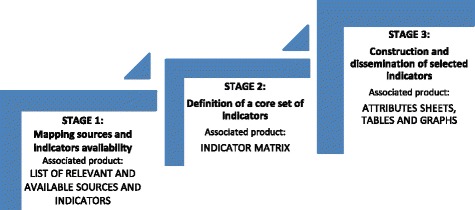
Three-stage methodology for the selection of sources, definition of a core set of indicators, and construction of selected indicators for an observatory on health inequities

Based on the Brazilian experience, a three-stage methodology^3^ could be envisioned for the development of HI observatories in LMIC to be implemented by their respective expert group on HI. We outline this methodology in Fig. 1, and will briefly describe each stage, emphasizing relevant steps for the creation of a Mexican observatory on HI. (A detailed description of the complete methodology is available elsewhere [17]). We consider this methodology to be innovative as it does not only provide specific guidance on the tasks to be carried out for measuring and analyzing HI, but it additionally links those major tasks with actions to develop public goods or “products” associated to each stage. This implies that the adequate progress of each stage of the development and functions of the observatory relates to an installed capacity to develop value-added products that aim to fulfil the ultimate goal of action on SDH, through the dissemination of information for health policies.

#### Stage 1: Mapping sources and availability of indicators

The possibilities of having already available larger sets of core indicators depend on each country’s tradition on producing statistical data on their information systems. Once the groups of experts leading the effort have decided on which relevant indicators for monitoring HI are to be included, the next step is to identify the data sources that contain such indicators. Generally speaking, information systems in LMIC tend to have fragmented data, imposing serious difficulties for calculating even the simplest indicators. For instance, whereas a sex ratio can be easily calculated using a single source (i.e. census data), calculating a global fertility rate may need the combination of two sources derived from two different information systems (i.e. census data and administrative records). This task becomes even more difficult when trying to identify HI indicators within countries, disaggregated at the smallest geographical units (i.e. municipalities, cities, etc.).

The mapping of sources and indicators includes: a) identification of already existent indicators; b) identification of relevant information systems for health and health related data for constructing new indicators and; c) identifying the lowest possible geographical level of disaggregation where the indicator preserves the attributes described above. As we have stressed earlier, an observatory on HI has a mandate to produce public goods. In this case, each stage of the methodology we describe is associated with a product for public use and with dissemination that can also be thought as a milestone for the corresponding phase. For this first stage, a list of relevant sources and available indicators may be presented to diverse sectors that can join in to collaborate in the health observatory initiative.

#### Stage 2: Definition of a core set of indicators

A crucial step for the creation of observatories on HI is the selection of stratifying variables that helps to assess whether a health disparity can be considered as an inequity. The selection of stratifying variables must be accompanied by strong and plausible associative assumptions with explanatory power. Examples of such variables are years of schooling, income, and social security affiliation. The selection should be based on a theoretical framework of social stratification, allowing assessing their impact on health.

As for the structural SDH, explanatory assumptions can be made based on a comprehensive analysis of societal characteristics that explain the processes in which these determinants interact with each other and have an impact on health. What follows, then, is the selection of summary indexes that can be used as proxy of such societal characteristics. For instance, the degree of inequality of income distribution can be measured using the Gross National Product *per capita* (GDPpc) or the Gini index, both indicators can be used as proxy of inequities. Another example is gender quotes in congresses seats which can be used as a proxy of gender equity. However, caution should be exercised when making the selection, as the explanatory power of a given indicator can change or disappear over time.

Once the indicators and their sources are mapped, they are gathered into thematic groups and systematized into a matrix where each category (i.e. sociodemographic, health status, health care, human resources for health) has its indicators described and exemplified (see example in Table 2). The matrix is the product that marks the finalization of this stage.

**Table 2:** Thematic groups of indicators for health inequities in Brazil (example)

GROUPS OF INDICATORS	EXAMPLES
General context and determinants of health	
Demographic	Proportion of elderly in the population by years of schooling and region of residence
Socioeconomic	Gross national product per capita by region
Living conditions	Proportion of population with access to sanitary sewage system by years of schooling and region of residence
Lifestyles	Prevalence of tobacco use by years of schooling and region of residence

Source: Brazilian Observatory on Health Inequities [28]

#### Stage 3: Construction and dissemination of selected indicators

This final stage refers to the documents and information to be disseminated to general audiences. As a basic rule, the information that the observatory should make available has to be clear and understandable, but also technically and methodologically precise in order to serve to the different purposes held by diverse audiences. Pairing the observatory with a web platform or portal, which complements formal periodic reports and contributes to the dissemination of evidence on SDH, has proved to be a successful mode of dissemination.

## Results and discussion

We have concisely described the methodology followed for the development of an OHI in LMIC, using as a guiding example the BOHI. In this section we will show some advances in applying this methodology for the Mexican context.

### Empirical basis in Mexico

Currently, in Mexico the positioning of the SDH is at the highest level of the health policy agenda. SDH are recurrently addressed in the Health Sector Program 2013–2018 (PROSESA) in its third chapter, and under the first main goal it is stated that there is a need: "To promote the participation of the public, social and private sectors to influence the social determinants of health" [23]. This and other similar statements throughout the document make reference to the importance of conducting actions to modify the SDH and confirm the existence of a window of opportunity to design and implement an observatory on SDH and HI in Mexico. In addition, Mexico also has a long tradition of health data availability and strong information systems (Table 3) which are of vital importance for the development of an OHI.

**Table 3:** Empirical basis for the creation of an observatory on SDH and HI in Mexico

Primary and specific information systems	Installed capacity at government organizations for publishing periodic reports on health and health-related issues	Installed capacities at the national decision-making level
• Primary information systems (name in parenthesis):	• National Population Council (CONAPO) • National Institute of Public Health (INSP) • Ministry of Social Development (SEDESOL) • National Institute of Statistics and Geography (INEGI) • National Council for the Evaluation of Social Development Policy (CONEVAL)	• Directorate General for Health Promotion • National Academy of Medicine • Observatory on Mental Health - Health Ministry of the State of Mexico • Observatory on Human Resources for Health • Observatory on Gender and Poverty • Directorate General of Performance Evaluation
o Census (INEGI)		
o Vital statistics and administrative records (INEGI)		
o Household surveys (INEGI)		
o Population estimates (CONAPO)		
• Specific information systems: National Health Information System (SINAIS):		
o Material and human resources from federal and state health ministries		
o Operational medical units in public health care		
o Financial accounting on health at federal and state level		
o Health care programs		
o Morbidity (hospital discharges in public health care by State)		
o Mortality (deaths): general, maternal and stillbirths		
o Birth certificate by year of event (2008–2012)		
CROSS-CUTTING COMPONENTS		
National Institute for Access to Public Information and Data Protection (INAI)		
Mexican Network on SDH (REDMEX-DSS)		
Mexican Foundation for Health (FUNSALUD)		

Nonetheless, there are capacities on SDH and HI research and management that are yet to be strengthened in the academia, research system, civil organizations, and decision making sectors in order to orient the topic of measuring health inequalities to achieve social justice and equity ([24]). This seems to indicate that the capacity of understanding and assessing HI among diverse sectors of society –who may conform the group of experts--, is growing at a slow pace, risking the opportunity to timely act on SDH.

In order to accelerate the development of the network and the consolidation of the expert group, we identified the Directorate General for Health Promotion of the Secretary of Health of Mexico (DGPS) and the Mexican Network on Social Determinants of Health (REDMEX-DSS) for the strengthening of linkages between several groups interested in the development of the observatory, using the momentum currently experienced with the positioning of the SDH in health policy planning for the next three years.

## Conclusion

In this contribution we have stressed the importance of measuring HI as recommended by the CSDH. We have also argued that the definition of health inequities has important practical consequences for its operationalization. The first step to approach the issue on measuring and monitoring HI is to theoretically underpin the notion of health inequities and strengthen health information systems that produce reliable statistical data on health disparities, which in turn can lead to the development of national observatories on HI. Although monitoring is certainly not sufficient to reduce health disparities, it can make an important contribution for improving accountability in public policy-making for action on SDH.

Using some principles of the methodology followed by the BOHI in the Mexican context we assess that the current political climate in Mexico offers an opportunity for the creation of an OHI. The positive fact that the federal government has a strong commitment in reducing HI could lead to the consolidation of an observatory. Nonetheless it remains the challenge of working under a collaborative scheme (such as the RIPSA agency) to avoid confusion, duplication of work, and unequitable distribution of financial resources between institutions.

The most important limitation we have found for implementing the methodology is that there is still a need to consolidate the experts group on HI that could apply and adopt the three-stage methodology, further testing its replicability. The identification of the empirical basis has helped us to disseminate the findings of this work among decision makers, researchers and civil society organizations in order to overcome the challenges and strengthen the linkages with other sectors.

Another important issue to consider is to envision adequate funding mechanisms for the eventual creation and sustainability of the Mexican observatory. In this concern, strategic alliances with policymakers and funding agencies are particularly relevant.

The greatest challenge is to integrate the sectors that are already working towards reaching equity in health, before the end of the current administration in 2018. Having an OHI in Mexico could help to gather all these different actors and maximize their participation to fulfill what the CSDH has acknowledged as a matter of death or life: social justice and equity within and between countries.

## Abbreviations

CSDH: Commission on Social Determinants of Health
HI: Health inequities
SDH: Social determinants of health
LMIC: Low and middle income countries
BOHI: Brazilian Observatory on Health Inequities
CEPI-DSS: Center for Studies, Policies and Information on Social Determinants of Health at Escola Nacional de Saúde Pública/ Fundação Oswaldo Cruz
OHI: Observatory on Health Inequities
GDPpc: Gross domestic product *per capita*
WHO: World Health Organization
NGO: Non- Governmental Organization
BNCSDH: Brazilian National Commission on Social Determinants of Health
RIPSA: Interagency Network for Health Information
PROSESA: Mexico’s Health Sector Program 2013-2018
SINAIS: National Health Information System
DGPS: Directorate General for Health Promotion of the Secretary of Health in Mexico
INEGI: National Institute of Statistics and Geography
CONAPO: National Population Council
CONEVAL: National Council for the Evaluation of Social Development Policy
REDMEX-DSS: Mexican Network on Social Determinants of Health
FUNSALUD: Mexican Foundation for Health

## Footnotes

1 We use “health inequalities” and “health disparities” as interchangeable terms for referring to measures in health between population groups without making associations or moral judgements on why these disparities are unjust, unfair, or avoidable [4].

2 In this sense it is worth to mention a recent effort by Garay [25] in conceptualizing and operationalizing this linkage by taking into account the “universal right to health” discourse, the World Health Organization (WHO) “health for all” principle, and the most influential variables of health status at population level into a single indicator: the “Health Inequity Burden”, defined as the difference between the current health situation of any given population group, and the best possible attainable health status present in countries whose life expectancy at birth is higher than the world’s average; their gross domestic product *per capita* (GDPpc) is lower than the world’s average; and their carbon footprint is below the planetary boundaries.

3 It should be noted that in 2013 the WHO published the “Handbook on health inequality monitoring: with a special focus on low- and middle-income countries” [26] The handbook derives from previous experiences of the WHO joint work with health ministries of LMIC for developing competencies in health inequality monitoring. It should be mentioned that the handbook not only emphasizes on the need for monitoring health inequalities in LMIC, but also presents a five step methodology (namely: 1) identify relevant health indicators; 2) obtain data about the indicators; 3) analyze the data; 4) report the results; and 5) implement changes) that aim to the improvement of policies on health inequities. The data used for the handbook is retrieved and published in the WHO’s Global Health Observatory: Health Equity Monitor [27]. The difference between the methodology presented by WHO and the one we propose here basically resides in the scope for approaching and taking on the challenges for a comprehensive monitoring of HI rather than the traditional epidemiological framing of health, based on disparities.
